# Is capsulectomy necessary for symptom improvement in patients undergoing implant removal for systemic symptoms?

**DOI:** 10.1080/23320885.2024.2390080

**Published:** 2024-08-21

**Authors:** Patricia McGuire, Caroline Glicksman

**Affiliations:** a Parkcrest Plastic surgery United States, United States; b Glicksman Plastic Surgery United States - Plastic Surgery, United States

**Keywords:** Case report, breast implants, capsulectomy

## Abstract

A case of symptom improvement after implant removal without capsulectomy is presented with a review of the literature.

Case report:

A 58-year-old woman presented requesting implant removal for systemic symptoms that she attributed to her smooth, saline, breast implants. The implants had been placed in a submuscular position in 2009 for aesthetic reasons (Figure 1). She has a history of diabetes and heart disease and her medications included metformin, Plavix (clopidogrel), and Wellbutrin (bupropion). She had developed a variety of symptoms in the preceding five years and had undergone and extensive work up by her primary care physician and a rheumatologist who failed to find any abnormality on physical exam or laboratory analysis. Out of frustration from a lack of a diagnosis for her symptoms, the patient searched the internet finding breast implant illness websites and social media groups that suggested her systemic symptoms were related to her breast implants. In addition, she found she had many of the same symptoms listed on these sites where women describe symptom improvement after explantation of their implants. Her board-certified plastic surgeon reviewed her medical records and with no other basis for her reported symptoms, she offered the patient implant removal. The surgical alternatives were discussed, including removal with or without capsulectomy along with the risks and benefits of each procedure. In light of the patient’s medical history, and medications including Plavix, there were concerns of a higher risk of post operative bleeding from a capsulectomy. The patient elected to undergo implant removal without capsulectomy. In the operating room she was found to have intact, smooth saline implants with translucent implant capsules (Figures 2 and 3). The implants were removed without capsulectomies or any modification to the capsule, such as cauterization or plication, and drains were not used. Three weeks after surgery, the patient’s only symptoms were weight gain, joint pain, and heartburn. At one year, her only reported symptom was occasional headaches. She was able to discontinue her Wellbutrin because of the improvement in her anxiety.

**Figure 1.**
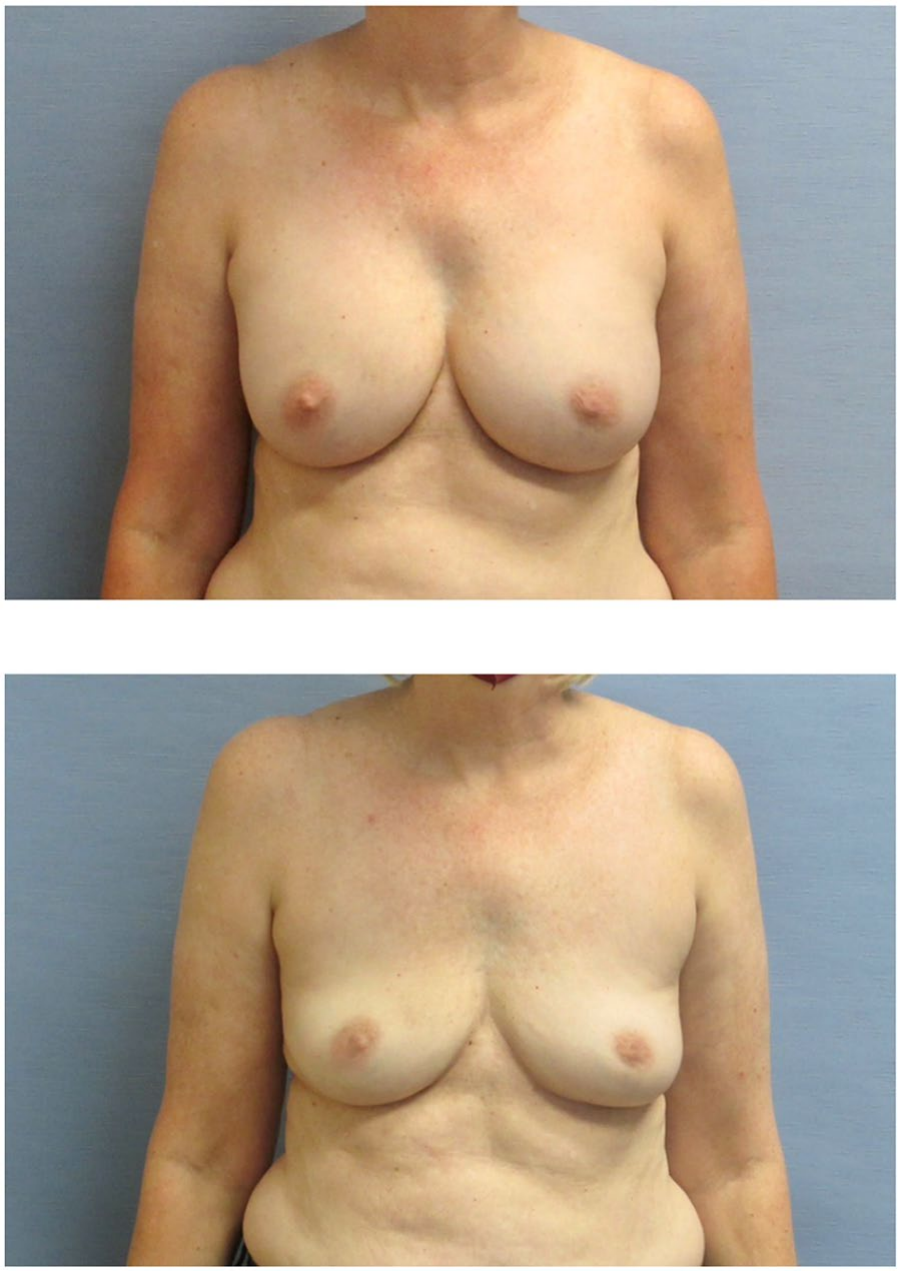
The patient before and 1 year after removal of her 300 cc smooth, saline implants.

**Figure 2.**
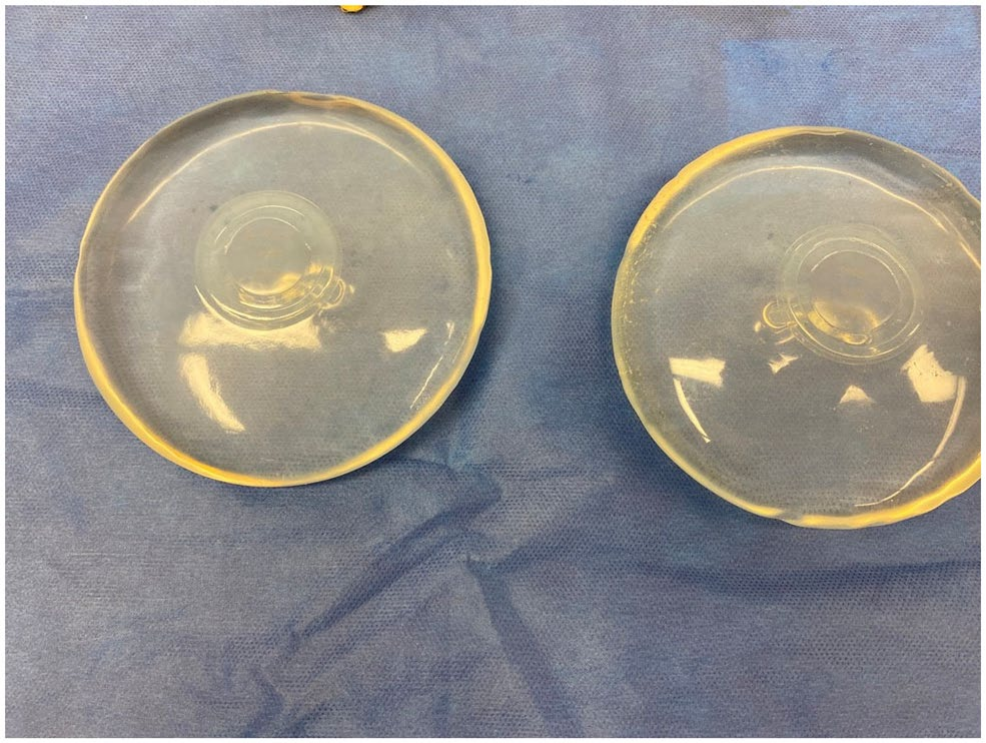
The removed, smooth, intact saline implants.

**Figure 3.**
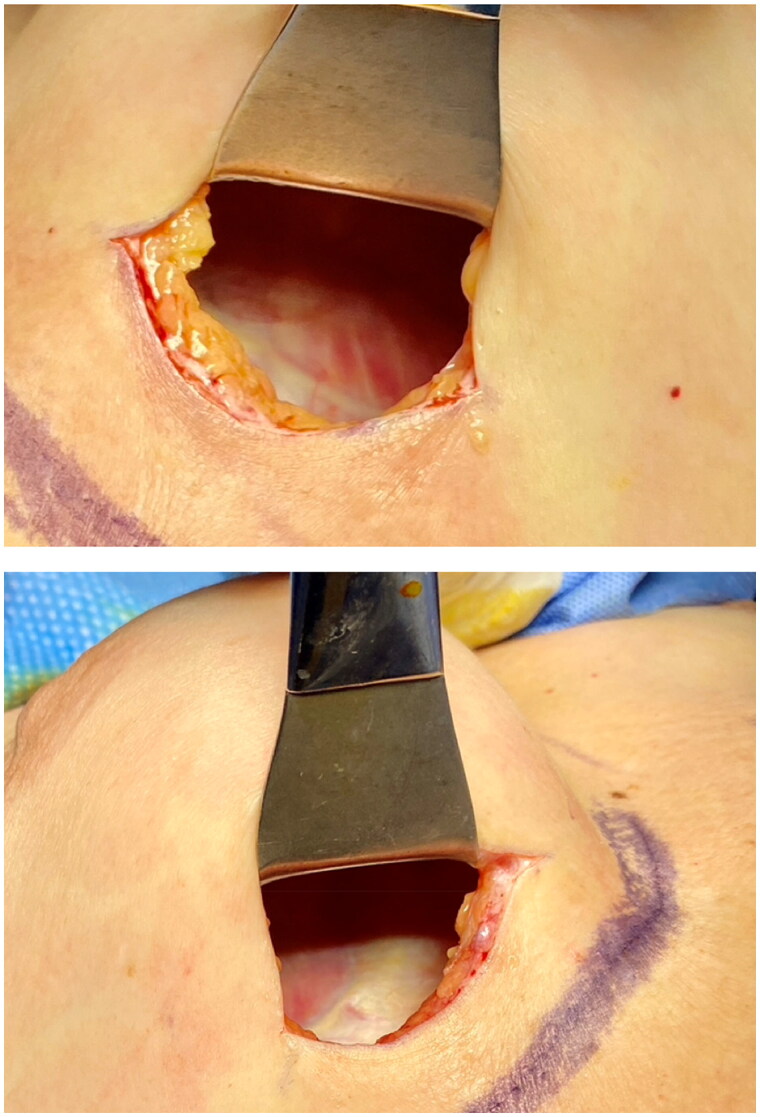
The translucent capsules.

Systemic symptoms associated with breast implants (SSBI), also referred to as breast implant illness (BII), refers to over one hundred of symptoms that patients attribute to their implants [1]. This has been reported with all implants by all manufacturers with no difference in incidence between saline, gel, smooth, or textured devices. These patients have no abnormal labs or physical findings to explain their symptoms and there are no specific diagnostic criteria. SSBI patients present to plastic surgeons requesting removal of not only the implant, but also the surrounding implant capsule. This request often stems from information they receive online and in social media groups where a “proper explant” is described as removing the implant and capsule together as a single unit, which they refer to as an “en bloc” capsulectomy. Their concern is that whatever toxins, such as heavy metals, silicone, and platinum that they believe are in the implant will also be in the implant capsule and leaving the capsule would leave those toxins behind which could interfere with symptom improvement [2].

The terminology used to describe capsulectomy has been inconsistent in the past making the discussion of risk and benefit difficult. We have suggested standardization in terminology for capsulectomy procedures as follows: en bloc capsulectomy refers to removal of an implant and capsule with a margin of uninvolved tissue used to treat capsular malignancy; total intact capsulectomy refers to removal of the implant and capsule as a single unit; total capsulectomy is the complete removal of the implant and capsule, not necessarily in one piece; partial capsulectomy refers to some capsule left behind; and no capsulectomy the entire capsule is left intact [3].

The only indication for an en bloc capsulectomy is malignancy of the capsule. The term, en bloc is therefore inappropriately used when referring to a procedure performed in the absence of capsular malignancy. The indications for total capsulectomy include grade 3 or 4 capsular contracture and intracapsular rupture of a gel implant to minimize gel extrusion outside of the capsule. Capsulectomy may be considered for removal or exchange of a textured device to smooth because of concerns of the potential for development of breast implant associated anaplastic large cell lymphoma (BIA-ALCL) or breast implant associated squamous cell carcinoma (BIA-SCC). There is currently no definitive data to show that capsulectomy is a risk reducing procedure for development of capsular malignancy, but a recent consensus paper stated that implant removal or exchange to smooth with or without a capsulectomy is reasonable in managing asymptomatic patients with textured implants [4]. The Food and Drug Administration (FDA) however, currently recommends against prophylactic implant removal in asymptomatic patients [4].

Previous studies have shown that patients with self-reported symptoms they attribute to their implants are likely to have at least partial symptom improvement after removal of their implants [5]. Studies showing symptom improvement with implant removal and total capsulectomy claim that capsulectomy is required at the time of implant removal for symptom improvement, but most of these studies are retrospective, have no long-term follow up, and lack a control group [6,7].

Capsulectomy is generally a more invasive procedure which takes longer to perform, thereby exposing the patient to a longer anesthetic time and potentially increased cost. It also requires a larger incision, and may carry a higher complication rate, specifically for hematoma. With an implant in a subpectoral position there is also the potential for development of a pneumothorax [8].

The Aesthetic Surgery Foundation Biospecimen Analysis Study was the first prospective, blinded study, with controls to evaluate symptom improvement in patients with SSBI. Eighty-eight percent of symptomatic subjects showed at least partial symptom improvement, with a 68% reduction in the number of symptoms reported through one year of follow up. The symptom improvement was independent of whether part of all the implant capsule was removed [5].

This study also evaluated capsules for heavy metals, bacterial and fungal DNA and histopathology. While some metals were detected in implant capsules of both subjects with self- reported breast implant illness and subjects without symptoms they attributed to their implants, all were in safe levels below concern. Metals were also found in breast tissue from a control group undergoing cosmetic mastopexy who never had any implanted medical device. There was no statistical difference in the type or levels of metals between all three study cohorts [9,10].

Analysis of capsules for the presence of bacteria or fungi using Next Generation Sequencing to identify the presence of bacterial and fungal DNA, showed no statistical difference in the presence of bacteria or any specific bacterial species between the cohorts. The only statistically significant difference was in the histologic examination of the capsules removed in both implant cohorts. There was higher incidence of synovial metaplasia in the SSBI cohort. This was associated with textured breast implants.

Detailed biospecimen analysis further included the evaluation of peripheral blood obtained from all three cohorts on the day of surgery. Specimens were tested for CBC, Thyroid levels, C-reactive protein, 12 different cytokines, and antibodies to Staph enterotoxins. All failed to show any consistent, measurable differences between the SSBI cohort and control cohorts.

In a prospective study from the Netherlands, BII subjects showed symptom improvement after undergoing implant removal. Seventy-eight percent of subjects had no capsule removed and showed symptom improvement equivalent to patients who had a capsulectomy performed [11].

A limitation of the Systemic Symptoms in Women Biospecimen Analysis Study was the lack of a no capsulectomy control. This was because in all subjects at least a partial capsulectomy was performed to provide tissue samples for bioanalysis per the study protocol. To answer this limitation, we performed another prospective study with a control group to follow systemic symptom improvement in SSBI subjects undergoing implant removal with no capsule tissue removed.

In the SSBI cohort, 54% of the implants were saline and 97% were smooth. The implants were in place for an average of 13.7 years and the average number of symptoms at baseline was 12.8. At the 3-6 week follow up visit, the number of symptoms had decreased to an average of 3.3 symptoms. This remained stable through 6 months of follow up.

The PROMIS data showed at baseline the SSBI cohort reported statistically higher levels of anxiety, fatigue, sleep disturbance, and lower cognitive function compared to the control group. At six months post op, all categories improved, with the biggest improvement in fatigue. Most significantly, the symptom improvement and PROMIS normalization seen in this study was statistically equivalent to what was seen in the previous study where patients underwent total and partial capsulectomy. Symptom improvement is likely after removal of implants in patients with systemic symptoms they associate with their breast implants, and the symptom improvement is independent of whether any capsule is removed [3].

The decision to perform a capsulectomy at the time of implant removal or exchange requires shared decision making between a surgeon and patient considering the risks and benefits. The factors that should be included in the discussion include the patient’s health and tolerance for a surgical procedure, whether the implant is intact or ruptured, the quality of the capsule, including the presence and severity of capsular contracture, and the type of implant, whether textured or smooth. Determination of a patient’s risk tolerance with the presence of a textured implant could also depend on whether the implant is macro or microtextured and the patient’s concern about the potential for the development of capsular malignancy. Any abnormality or suspected abnormality of the capsule requires preoperative evaluation. Patients with systemic symptoms they associate with their implants should have a thorough medical evaluation prior to undergoing and surgery to rule out other medical etiologies.

There are surgeons who promote themselves as “en bloc” capsulectomy experts and claim to possess special surgical skills that are lacking in other board-certified plastic surgeons with no evidence to back these claims [12]. Patients who consult with these surgeons may only be given the option of implant removal with a total capsulectomy. Patients have a right to have their implants removed and to request a capsulectomy. The procedure, however performed, should be selected after an educated discussion between the patient and her surgeon based on published, peer reviewed scientific data, so that she can make the best decision for her individual circumstance.
